# Prevalence of SARS-CoV-2 Antibodies in First Responders and Public Safety Personnel, New York City, New York, USA, May–July 2020

**DOI:** 10.3201/eid2703.204340

**Published:** 2021-03

**Authors:** Samira Sami, Lara J. Akinbami, Lyle R. Petersen, Addie Crawley, Susan L. Lukacs, Don Weiss, Rebecca A. Henseler, Nga Vuong, Lisa Mackey, Anita Patel, Lisa A. Grohskopf, Beth Maldin Morgenthau, Demetre Daskalakis, Preeti Pathela

**Affiliations:** Centers for Disease Control and Prevention, Atlanta, Georgia, USA (S. Sami, A. Patel, L.A. Grohskopf);; Centers for Disease Control and Prevention, Hyattsville, Maryland, USA (L.J. Akinbami, S.L. Lukacs);; US Public Health Service, Rockville, Maryland, USA (L.J. Akinbami, S.L. Lukacs, L.A. Grohskopf);; Centers for Disease Control and Prevention, Fort Collins, Colorado, USA (L.R. Petersen, N. Vuong, L. Mackey);; New York City Department of Health and Mental Hygiene, Queens, New York, USA (A. Crawley, D. Weiss, R.A. Henseler, B. Maldin Morgenthau, D. Daskalakis, P. Pathela)

**Keywords:** seroepidemiologic studies, emergency responders, personal protective equipment, New York City, New York, United States, public safety, 2019 novel coronavirus disease, SARS-CoV-2, severe acute respiratory syndrome coronavirus 2, coronavirus disease, COVID-19, viruses, respiratory infections, zoonoses

## Abstract

We conducted a serologic survey in public service agencies in New York City, New York, USA, during May–July 2020 to determine prevalence of severe acute respiratory syndrome coronavirus 2 (SARS-CoV-2) infection among first responders. Of 22,647 participants, 22.5% tested positive for SARS-CoV-2–specific antibodies. Seroprevalence for police and firefighters was similar to overall seroprevalence; seroprevalence was highest in correctional staff (39.2%) and emergency medical technicians (38.3%) and lowest in laboratory technicians (10.1%) and medicolegal death investigators (10.8%). Adjusted analyses demonstrated association between seropositivity and exposure to SARS-CoV-2–positive household members (adjusted odds ratio [aOR] 3.52 [95% CI 3.19–3.87]), non-Hispanic Black race or ethnicity (aOR 1.50 [95% CI 1.33–1.68]), and severe obesity (aOR 1.31 [95% CI 1.05–1.65]). Consistent glove use (aOR 1.19 [95% CI 1.06–1.33]) increased likelihood of seropositivity; use of other personal protective equipment had no association. Infection control measures, including vaccination, should be prioritized for frontline workers.

Coronavirus disease (COVID-19) was recognized in New York City (NYC), New York, USA, in late February 2020 and had spread throughout the community by March 2020 (*1*). First responders and public safety personnel have played a critical role in the COVID-19 pandemic response. Understanding the occupational risks for severe acute respiratory syndrome coronavirus 2 (SARS-CoV-2) infection is vital for designing workplace prevention protocols to reduce transmission. Serologic surveys can identify the prevalence of previous SARS-CoV-2 infection in the population.

We conducted a serologic survey to estimate SARS-CoV-2 infection prevalence among first responders, public safety personnel, and other public service workers in NYC. The study objectives were to determine the prevalence of IgG against SARS-CoV-2 and to examine associations between characteristics and occupational exposures and previous infection among workers in emergency response and public safety settings.

## Methods

This cross-sectional survey was conducted during May 18–July 2, 2020, in the 5 NYC boroughs: Brooklyn, Manhattan, Queens, Staten Island, and the Bronx. The Institutional Review Board of the NYC Department of Health and Mental Hygiene and Centers for Disease Control and Prevention (CDC) human subjects research officials determined this activity to be public health surveillance as defined in 45 CFR 46.102(l) (*2*).

Adults >18 years of age working onsite in a public service agency were eligible to participate, including employees of city departments of corrections, police, fire, medical examiner, and education, for a total of ≈60,000 persons. Educational settings were limited to Regional Enrichment Centers that served children of first responders and healthcare personnel. Persons who self-reported a positive result for SARS-CoV-2 or occurrence of COVID-19 symptoms <2 weeks before completing the questionnaire were ineligible.

A questionnaire assessed participant demographics and relevant household, occupation, and workplace risk factors for SARS-CoV-2 infection (Appendix Table 1). Participation was voluntary. Consenting participants completed the questionnaire online and provided a blood specimen at a collection site located at or near their workplace during May 18–July 2, 2020. Samples were tested for SARS-CoV-2 antibodies by using the VITROS Immunodiagnostic Products Anti-SARS-CoV-2 IgG Test (ORTHO Clinical Diagnostics Inc., https://www.orthoclinicaldiagnostics.com). Data for this test submitted to the Food and Drug Administration indicated a sensitivity of 90% and a specificity of 100% (*2*). Some tests were not performed because of lipemia or insufficient serum. CDC did not receive personal identifiers, and individual results were not shared with employers.

Participants self-reported their race or ethnicity. Reported height and weight were used to calculate body mass index (BMI); weight status categories were defined as underweight or normal (BMI <25), overweight (BMI >25 but <30), obese (BMI >30 but <40), and severely obese (BMI >40). Nonhospital healthcare workers (physicians, midlevel clinicians, nurse assistants, nurses, therapists, phlebotomists, imaging technicians, and dentists) were categorized as other direct patient care providers. Frequency of use of personal protective equipment (PPE) within 6 feet of a person with suspected or confirmed COVID-19 was categorized as all of the time, not all of the time (never or rarely, sometimes, and most of the time), and not applicable.

A total of 22,647 participants were included in our analysis (Appendix Figure 1). Percentage of SARS-CoV-2 IgG seropositivity and 95% CIs were calculated by selected characteristics and exposures. In subsequent analyses assessing seropositivity by frequency of aerosol-generating procedures and PPE use, we focused on occupations for which CDC-issued recommendations for PPE were in place: police (including traffic officers), medicolegal death investigators, firefighters, correctional staff, security guards, firefighters or medical first responders, paramedics, emergency medical technicians (EMTs), dispatchers (fire, emergency medical service [EMS], or police), and other direct patient-care providers (*3*–*6*). We performed multivariable logistic regression with seropositivity as the outcome variable. Covariates were chosen a priori and checked for collinearity. Participants with implausible weight or height (n = 15) or missing housing status (n = 6) were excluded. We used SAS version 9.4 (SAS Institute, https://www.sas.com) to perform statistical analyses. We considered 2-sided p values <0.05 to be statistically significant.

## Results

A total of 5,091 (22.5% [95% CI 21.9%–23.0%]) participants tested positive for SARS-CoV-2 IgG (Table); however, only 10.1% (95% CI 9.8%–10.5%]) of participants reported previous positive results for SARS-CoV-2 by reverse transcription PCR. Seroprevalence was higher among women than men, higher among non-Hispanic Black persons than other racial or ethnic groups, higher among persons 18–24 years of age compared with older age groups, and higher among persons who were severely obese compared with those with a lower weight status (Table). Seropositivity was highest among those with exposure to a household member who tested positive for SARS-CoV-2 (48.3% [95% CI 46.3%–50.3%]). In addition, seropositivity was highest among persons who resided in the Bronx (28.8% [95% CI 26.8%–30.9%]) and lowest among those residing outside of NYC (18.3% [95% CI 17.5%–19.2%]). Participants who lived in multiunit housing had higher seropositivity than those who lived in single-family housing, as did participants in very large households (>8 persons) compared with households of <7 persons (Appendix Figure 2).

**Table:** Percentage of respondents who were seropositive for SARS-CoV-2 IgG, by demographic and health characteristics, in a study of first responders and public safety personnel, New York City, New York, USA, May 18–July 2, 2020*

Characteristic	No. (%)		% Seropositive (95% CI)	
Total	22,647 (100.0)		22.5 (21.9–23.0)	
Sex				
M	17,118 (75.6)		21.9 (21.3–22.5)	
F	5,529 (24.4)		24.2 (23.1–25.4)	
Age group, y				
18–24	795 (3.5)		32.0 (28.7–35.3)	
25–34	6,677 (29.5)		26.4 (25.3–27.5)	
35–44	8,034 (35.5)		20.2 (19.4–21.1)	
45–59	6,328 (27.9)		20.3 (19.4–21.4)	
60–64	589 (2.6)		20.7 (17.5–24.2)	
>65	224 (1.0)		18.3 (13.5–24.0)	
Race/ethnicity				
Non-Hispanic White	10,013 (44.2)		18.5 (17.7–19.2)	
Non-Hispanic Black	3,292 (14.5)		30.1 (28.5–31.7)	
Non-Hispanic Asian	1,647 (7.3)		21.3 (19.4–23.4)	
Hispanic or Latino	5,460 (24.1)		26.9 (25.7–28.1)	
Non-Hispanic other race†	548 (2.4)		20.3 (17.0–23.9)	
Decline to answer	1,687 (7.5)		19.3 (17.4–21.2)	
Weight status, n = 22,632‡				
Underweight or normal weight	4,048 (17.9)		21.4 (20.2–22.7)	
Overweight	10,386 (45.9)		22.1 (21.3–22.9)	
Obese	7,500 (33.1)		23.1 (22.1–24.1)	
Severely obese	698 (3.1)		27.8 (24.5–31.3)	
Housing, n = 22,641				
Single family	15,455 (68.3)		21.1 (20.5–21.8)	
Multiunit	7,186 (31.7)		25.3 (24.3–26.4)	
Residence borough				
Outside New York City	8,654 (38.2)		18.3 (17.5–19.2)	
Bronx	1,948 (8.6)		28.8 (26.8–30.9)	
Brooklyn	3,329 (14.7)		28.0 (26.5–29.5)	
Manhattan	1,207 (5.3)		21.4 (19.1–23.8)	
Queens	4,834 (21.3)		25.4 (24.2–26.6)	
Staten Island	2,675 (11.8)		19.8 (18.3–21.3)	
Workplace§				
Correctional facility	1,272 (5.6)		36.2 (33.6–39.0)	
Emergency medical services	2,418 (10.7)		35.2 (33.3–37.2)	
Childcare setting (Regional Enrichment Center)	677 (3.0)		21.3 (18.2–24.6)	
Fire services	6,087 (26.9)		20.8 (19.8–21.9)	
Police department	11,885 (52.5)		19.8 (19.1–20.5)	
Medical examiner office	394 (1.7)		11.7 (8.7–15.3)	
Workplace borough				
Bronx	3,524 (15.6)		26.8 (25.4–28.3)	
Brooklyn	6,075 (26.8)		24.1 (23.1–25.2)	
Manhattan	5,755 (25.4)		19.7 (18.6–20.7)	
Queens	6,200 (27.4)		21.9 (20.9–23.0)	
Staten Island	1,093 (4.8)		17.4 (15.2–19.8)	
Exposure to persons who tested positive for SARS-CoV-2‡				
Household member	2,393 (10.6)		48.3 (46.3–50.3)	
Coworker	14,912 (65.9)		23.7 (23.0–24.3)	
Patient	6,502 (28.7)		26.9 (25.8–28.0)	
Other person	7,721 (34.1)		26.8 (25.8–27.8)	

*BMI, body mass index; SARS-CoV-2, severe acute respiratory syndrome coronavirus 2. †Non-Hispanic other race includes Native Hawaiian and other Pacific Islander and American Indian and Alaska Native. ‡Weight status categories defined as underweight or normal weight (BMI <25), overweight (BMI >25 but <30), obese (BMI >30 but <40), and severely obese (BMI >40). §Workplace and self-reported exposure to persons who tested positive for SARS-CoV-2 are not mutually exclusive.

**Figure 1:**
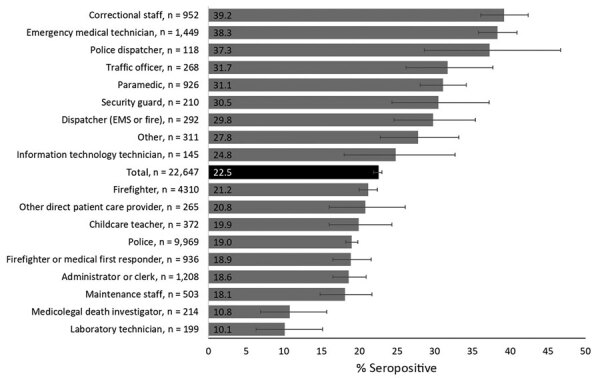
Percentage of respondents who were seropositive for severe acute respiratory syndrome coronavirus 2 IgG, by occupation, in a study of first responders and public safety personnel, New York City, New York, USA, May 18–July 2, 2020. Numbers within bars indicate percentage of seropositive respondents. Error bars indicate 95% CIs. Other includes students or trainees, pharmacists, medical registrars, orderlies, dietitians, medical assistants, counselors, social workers, dietary services staff, environmental services staff, and participants who selected this category and were not reassigned to an existing category. Firefighters includes fire inspectors and fire marshals. Other direct patient care providers include dentists, diagnostic imaging technicians, midlevel clinicians, nurses, nurse assistants, occupational therapists, speech therapists, physical therapists, phlebotomists, physicians, respiratory therapists, and therapy aides. EMS, emergency medical service.

Seroprevalence was higher among those who worked in correctional facilities (36.2% [95% CI 33.6%–39.0%]) and EMS agencies (35.2% [95% CI 33.3%–37.2%]) compared with those who worked in other workplaces (range 11.7%–21.3%) (Table). Seroprevalence also varied by occupation (Figure 1). We also observed differences in seroprevalence by workplace borough; prevalence was highest in the Bronx (26.8%) and lowest in Staten Island (17.4%) (Table).

**Figure 2:**
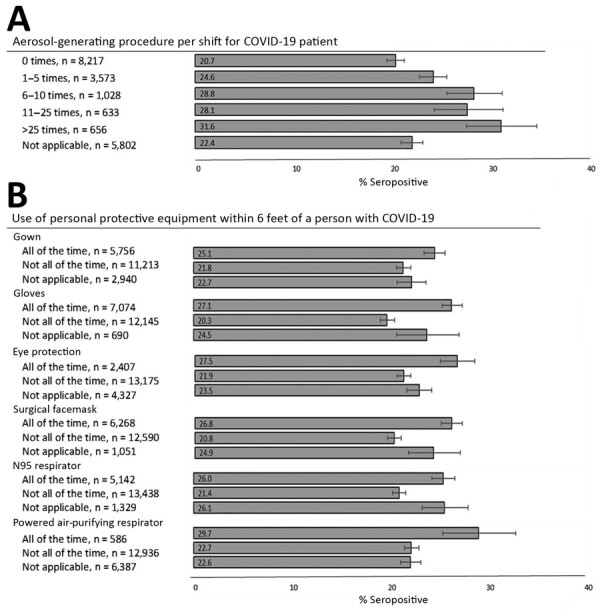
Unadjusted percentage of respondents who were seropositive for severe acute respiratory syndrome coronavirus 2 IgG, by aerosol-generating procedure frequency (A) and use of personal protective equipment (B), in a study of first responders and public safety personnel, New York City, New York, USA, May 18–July 2, 2020. Numbers within bars indicate percentage of seropositive respondents. Error bars indicate 95% CIs. First responders and public safety personnel include police, medicolegal death investigators, firefighters, correctional staff, security guards, traffic officers, police dispatchers, firefighters or medical first responders, paramedics, emergency medical technicians, dispatchers (emergency medical service or fire), and other direct patient-care providers. COVID-19, coronavirus disease.

The remainder of the analysis focused on first responders and public safety personnel (n = 19,909) (*3*–*6*). Seropositivity increased with increasing frequency of aerosol-generating procedures performed per shift (p = 0.002), ranging from 20.7% among persons who did not conduct these procedures to 31.6% among those who conducted procedures >25 times on average per shift (Figure 2). Seropositivity also varied by frequency of PPE use when within 6 feet of a person with confirmed or suspected COVID-19, including stratification by occupation (Figure 2; Appendix Figure 3). Overall, for each PPE component, those who reported use all of the time had a significantly higher percent positivity than those who reported not all of the time (p<0.05).

**Figure 3:**
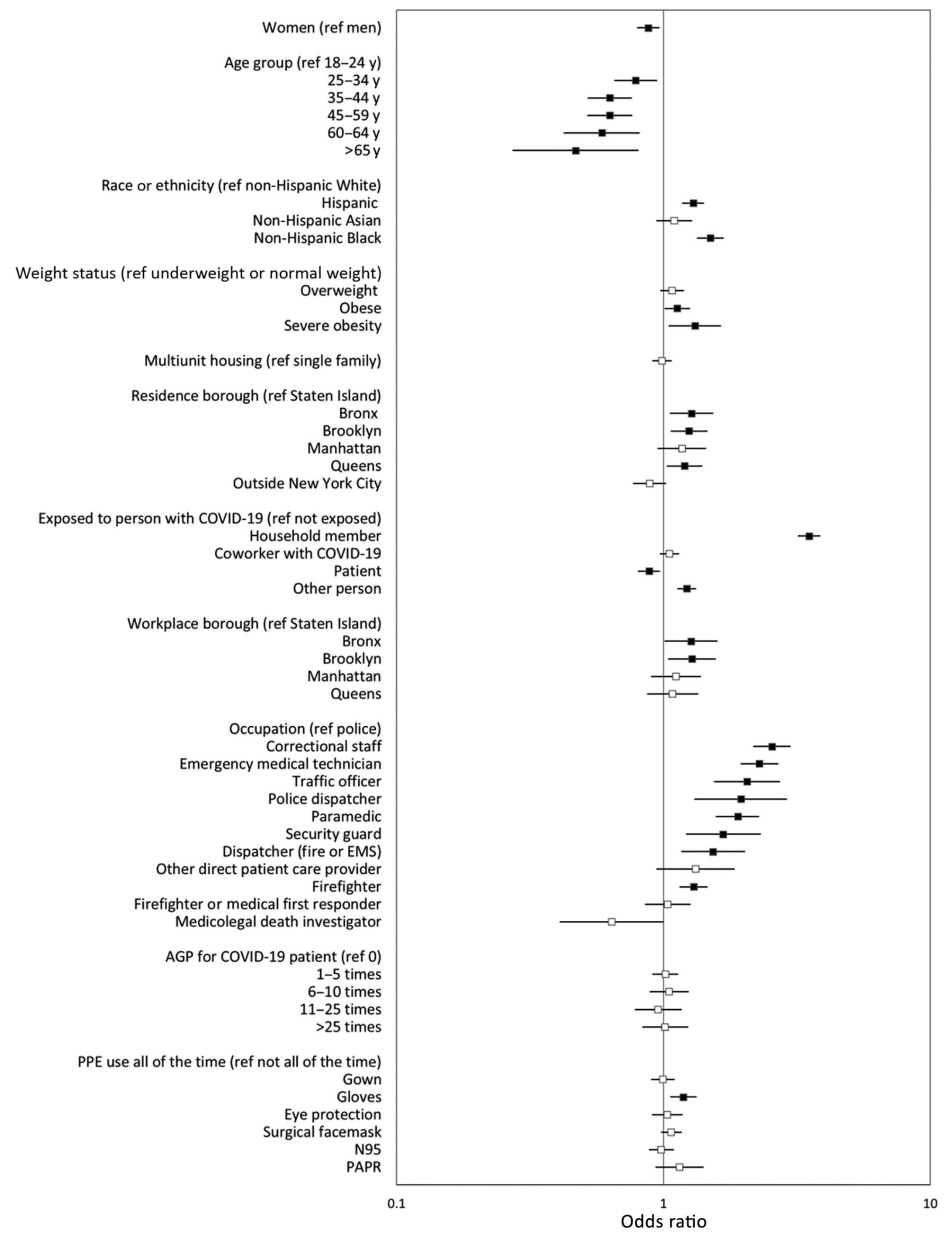
Adjusted odds ratios of seropositivity for severe acute respiratory syndrome coronavirus 2 IgG in a study of first responders and public safety personnel, New York City, New York, USA, May 18–July 2, 2020. Adjusted model includes all variables shown. Black boxes indicate statistically significant results; error bars indicate 95% CIs. Participants of other racial or ethnic groups or who declined to provide their race or ethnicity are included in the models but not shown as separate categories. Variables for exposure to person with COVID-19 are not mutually exclusive. AGP, aerosol-generating procedure; COVID-19, coronavirus disease; EMS, emergency medical service; ref, referent; PAPR, powered air-purifying respirator; PPE, personal protective equipment.

In adjusted analyses, women and those exposed to a patient with suspected or confirmed COVID-19 were less likely to be seropositive than their counterparts (Figure 3; Appendix Table 2). Characteristics associated with increased odds of seropositivity were self-reported exposure to a household member who tested positive for SARS-CoV-2, non-Hispanic Black versus non-Hispanic White race or ethnicity, severe obesity versus underweight or normal weight status, and residing or working in Brooklyn versus Staten Island. Correctional staff, EMTs, traffic officers, paramedics, security guards, dispatchers (EMS or fire and police), and firefighters were more likely than police to be seropositive; correctional staff had the highest likelihood of seropositivity (adjusted odds ratio [aOR] 2.55 [95% CI 2.18–2.99]). The aOR for seropositivity when using any PPE component all of the time was not significant. However, workers who reported using gloves all of the time were significantly more likely than those who used gloves not all of the time to be seropositive (aOR 1.19 [95% CI 1.06–1.33]).

## Discussion

SARS-CoV-2 seroprevalence among public service agencies personnel (22.5%) was similar to the 19.5% seroprevalence estimate for NYC residents during comparative dates (*7*). However, seroprevalence varied nearly 4-fold by occupation, ranging from 10.1% in laboratory technicians to 39.2% in correctional staff. Similar to other studies, we found seroprevalence varied by nonoccupational factors such as race or ethnicity, age group, weight status, housing type, residence borough, and exposure to household members with COVID-19 (*8*; J.M. Baker, unpub. data, https://doi.org/10.1101/2020.10.30.20222877). However, even when controlling for these factors, we found that seroprevalence for police and firefighters was close to that of the general population; conversely, correctional staff and EMTs, the occupations with the highest seropositivity in our study, had a seroprevalence twice as high (*7*). These populations face unique challenges when working in congregate or uncontrolled settings and would be a critical population for vaccination and other public health efforts to reduce SARS-CoV-2 infection.

Correctional facility workers had the highest seroprevalence of SARS-CoV-2 antibodies, and the odds of seropositivity were more than double for these workers compared with police, a group with a seroprevalence similar to the general population. COVID-19 in congregate settings has spread rapidly because of crowded living conditions and few options for isolation of exposed persons (*9*–*11*). Recent data from mass testing in correctional facilities found SARS-CoV-2 prevalence ranged from 0% to 87% (*12*). In New York state, 3,762 COVID-19 cases had been reported among staff of 28 correctional and detention facilities as of September 6, 2020 (*13*). Such recommendations as grouping persons with laboratory-confirmed infection are crucial to prevent COVID-19 outbreaks in correctional facilities, but additional strategies are needed for settings in which isolating multiple persons infected with SARS-CoV-2 might not be possible (*5*,*14*).

Among healthcare workers, EMTs had a seroprevalence of 38.3% and the strongest association with seropositivity after adjustment. In contrast, other SARS-CoV-2 studies among NYC hospital-based healthcare workers found a seroprevalence ranging from 14% to 27% (*15*,*16*). EMS often occur in uncontrolled, unpredictable environments in which space is limited (e.g., ambulances) and require rapid decisions that might increase employee exposure risk. Although EMTs and paramedics both conduct procedures with a high risk for exposure (e.g., airway management), paramedics had a significantly lower seroprevalence than EMTs (*17*). Unmeasured factors, such as level of training, might account for the higher likelihood of seropositivity among EMTs compared with paramedics, who undergo an additional >1,000 training hours (*18*).

Other occupations with notably elevated seropositivity included traffic officers, security guards, and emergency dispatchers. Persons in these occupations have frequent and close interactions with the general public or work in environments in which space between coworkers is limited. Conversely, medicolegal death investigators and laboratory technicians, occupations with the lowest seroprevalence, might have less frequent close contact with other persons. Our findings also suggest that infection rates in the workplace might correlate with underlying community transmission, and not all observed associations are consistent with occupational risk. After adjustment, persons who worked or resided in the Bronx or Brooklyn had higher seropositivity compared with persons who worked or lived in Staten Island. This finding aligns with test results reported to the NYC Department of Health and Mental Hygiene, which found higher community seroprevalence in the Bronx (32.2%) and Brooklyn (27.0%) than in Staten Island (19.6%) (*1*).

Our finding that consistent use of gloves was associated with seropositivity was unexpected. However, among occupations without extensive training in glove use, a paradoxical association with infection has been previously observed: higher infection rates among consistent glove users was caused by cross-contamination and lack of hand hygiene after glove removal (*19*–*22*). PPE use has been demonstrated to be effective among healthcare workers in facility settings, but our study of first responders and public safety personnel in nonfacility settings demonstrates a different pattern, which warrants further investigation (*23*). Studies among healthcare workers found improper use of PPE, insufficient training, and perceived inadequacy of supplies increased transmission of other coronaviruses and might explain the higher seroprevalence documented in our study (*24*–*26*). Greater PPE use might be a surrogate for greater exposure to COVID-19 in the workplace. According to the hierarchy of controls, engineering and administrative controls (e.g., isolation and indoor ventilation) are preferred, and PPE should be the last line of defense to protect workers (*27*).

Public service personnel exposed to a SARS-CoV-2–positive household member also had higher seropositivity, a finding consistent with another study (*28*). This finding indicates the importance of managing exposure risk within households of frontline workers. Another factor to consider in NYC is the high density of living conditions, which was associated with greater likelihood of infection in our study. Even after controlling for occupation and housing type, racial and ethnic minority groups had higher seropositivity than non-Hispanic White workers. This pattern might be explained by unmeasured social disparities, such as lower income status, lack of paid sick leave, and mass transit use, which have been found to be associated with seropositivity among racial and ethnic minority groups in NYC (*29*; D. Carrion, unpub. data, https://doi.org/10.1101/2020.06.02.20120790; K.T.L Sy, unpub. data, https://doi.org/10.1101/2020.05.28.20115949). Mitigation measures should address persons working or residing in areas with high levels of SARS-CoV-2 transmission and racial or ethnic disparities.

Limitations of our study include that it was a convenience sample of public service agency personnel with limited numbers of healthcare professionals; participation ranged from an estimated 11% of ≈11,600 eligible correctional facility personnel to 81% of ≈10,300 fire services personnel. Participation might have been influenced by prior results of testing by reverse transcription PCR, expanded access to free antibody testing in the city, household exposure, and worker availability. Data collection occurred during May 18–July 2, 2020; recall bias could have affected responses for exposures 3 months before the survey. Study participants were also asked to recall PPE use during a wide period, and questions were not designed to measure adaptation to evolving PPE use. Temporality also limits our ability to know whether infection occurred before or after a potential exposure. Despite these limitations, our study provides seroprevalence estimates and factors associated with SARS-CoV-2 infection across a diverse set of occupations for which little data exist.

Nearly 25% of first responders and public safety personnel in our study were infected with SARS-CoV-2 before July 2020. Seroprevalence varied by nearly 4-fold among occupations; correctional staff and EMTs demonstrated highest levels of seropositivity. Other occupations with frequent close contact with the public also had elevated seroprevalence. We did not observe lower seroprevalence levels as expected from self-reported consistent PPE use, possibly because persons with consistent use had higher and more frequent exposure to SARS-CoV-2. Nevertheless, these results have identified high-risk occupations for which enhanced prevention measures including engineering and administrative controls and vaccination are required.

Appendix Additional information about prevalence of SARS-CoV-2 antibodies in first responders and public safety personnel, New York City, New York, USA, May–July 2020.
