# Supplementary material for: Prevalence of SARS-CoV-2 Antibodies in First Responders and Public Safety Personnel, New York City, New York, USA, May–July 2020
Source: Emerg Infect Dis. 2021 Mar;27(3):796–804. doi: 10.3201/eid2703.204340 (PMC7920688; doi:10.3201/eid2703.204340)
Supplement: Appendix — Additional information about prevalence of SARS-CoV-2 antibodies in first responders and public safety personnel, New York City, New York, USA, May–July 2020. [file 20-4340-Techapp-s1.pdf]

Article DOI: https://doi.org/10.3201/eid2703.204340

Prevalence of SARS-CoV-2 Antibodies in First Responders and Public Safety Personnel, New York City, New York, USA, May–July 2020

Appendix

Appendix Table 1. Survey questionnaire administered in study of first responder and public safety personnel, New York City, New York, USA, May 18–July 2, 2020*

Question no.	Questionnaire item	Response categories
1.	Name (not provided to CDC)	First<br>Last
2.	Home address (not provided to CDC)	Street<br>City<br>State<br>Zip
3.1	Phone number (mobile) (not provided to CDC)	Area code, phone number
3.2	Verify phone number (mobile) (not provided to CDC)	Area code, phone number
4.1	Email address (not provided to CDC)	(fill)
4.2	Verify email address (not provided to CDC)	(fill)
5.	County/borough of workplace	[scroll] (jurisdictions to provide list)<br>Other (fill)
6.	Date of birth (not provided to CDC)	MM/DD/YYYY
7.1	Sex at birth	Male<br>Female
7.2	Current gender (not provided to CDC)	Man<br>Woman<br>Transgender man/trans man/female-to-male (FTM)<br>Transgender woman/trans woman/male-to-female (MTF)<br>Genderqueer/gender nonconforming neither exclusively male nor female<br>Other (fill)<br>Decline to answer
8.	Sexual orientation (not provided to CDC)	Gay<br>Straight<br>Bisexual<br>Something else/not sure<br>Decline to answer
9.	Are you Hispanic or Latino/Latina?	Yes<br>No<br>Don't know<br>Decline to answer
10.	What is your race? (select all that apply)	White<br>Black/African American<br>Asian<br>American Indian or Alaska Native<br>Native Hawaiian or other Pacific Islander<br>Other<br>Decline to answer
10A	Please select your age group	18–29 y<br>30–39 y<br>40–49 y<br>50–59 y<br>60–64 y<br>65–69 y

Question no.	Questionnaire item	Response categories
		70 y or older
11.1	What is your occupation?	Nurse (e.g., RN, APRN, LPN)<br>Midlevel clinician (e.g., PA, NP, nurse-midwife)<br>Nurse assistant (e.g., CNA)<br>Physician (e.g., MD, DO)<br>Student or trainee<br>Medical examiner<br>Mortuary technician<br>Police officer<br>Firefighter<br>Paramedic<br>Emergency medical technician<br>Medical first responder<br>Corrections officer or corrections staff<br>Respiratory therapist<br>Occupational/physical/speech therapist<br>Therapy aide/assistant<br>Pharmacist, pharmacist assistant<br>Diagnostic imagining technologist/technician<br>Clinical laboratory technologist/technician<br>Phlebotomist<br>Social worker/case manager<br>Administration, clerk<br>Medical records specialist/medical registrar<br>Dietary services staff<br>Environmental services staff<br>Maintenance staff<br>Orderly/transportation staff<br>Security guard
12.1	What is your main workplace or work setting? (select all that apply) (skip question 12.2 if non-hospital response)	Emergency department<br>Hospital intensive care unit<br>Hospital ward/floor<br>Other hospital location<br>Fire station/department<br>Police station/department<br>Correctional facility<br>Medical examiner office<br>Other (fill)
12.2	What hospital do you work at?	[scroll] (jurisdictions to provide list)<br>Other (fill)
13.1	Since March 1st, how many weeks have you worked in this setting?	Weeks (scroll) [min of 1, max of 20]
13.2	Since March 1st, on average, how many shifts did you work per week in this setting?	Shifts (fill) [min of 1, max of 14]
13.3	Since March 1st, on average, how many hours did you work per shift?	Hours (scroll) [min of 1, max of 24]
15.1	In the course of your work, how often did you use personal protective equipment (PPE) when within 6 feet of a person with suspected or confirmed COVID-19? All the time; Most of the time; Sometimes; Rarely/never; Not applicable	Gown (Go to Q 18)
15.2		Gloves (Go to Q 18)
15.3		N95 respirator (Go to Q 16)
15.4		Powered air purifying respirator (PAPR) (Go to Q 17)
15.5		Goggles or face shield (Go to Q 18)
15.6		Surgical facemask (Go to Q 18)
15.7		Other respirator (Go to Q 18)
16.	In the last year, have you been fit tested for the respirator you wore?	Yes<br>No<br>Don't know
17.	In the last year, have you been trained to use the respirator you wore?	Yes<br>No<br>Don't know
18.	Since March 1, on average, how many times per shift did you participate in any aerosol-generating procedures for suspected or confirmed COVID-19 patient(s)? For example,	More than 25 times

Question no.	Questionnaire item	Response categories
	open suctioning of airways; sputum induction; cardiopulmonary resuscitation; endotracheal intubation and extubation; noninvasive ventilation (e.g., BiPAP, CPAP); bronchoscopy; manual ventilation.	11–25 times<br>6–10 times<br>1–5 times<br>0<br>Not applicable
19.1	Did you spend more than 10 min within 6 feet of a coworker who tested positive for COVID-19?	Yes<br>No<br>Don’t know
19.2	Did you spend more than 10 min within 6 feet of a household member who tested positive for COVID-19?	Yes<br>No<br>Don’t know
19.3	Did you spend more than 10 min within 6 feet of any other person who tested positive for COVID-19?	Yes<br>No<br>Don’t know
20	How many times were you tested for COVID-19? (nasal, throat, or saliva sample)	(scroll) [indicate 0 if none] (if 0 Go to 23.1)
21.	Have you ever had a positive result for COVID-19?	Yes<br>No<br>Don’t know
22.	Approximately, when was the last time you were tested for COVID-19 in 2020 (excluding blood testing for exposure to SARS-CoV-2)?	MM/DD<br>Don’t know
23.1	Since March 1, have you experienced any of the following symptoms?	Fever<br>Chills<br>Cough (new onset or worsening of chronic cough)<br>Sore throat<br>Shortness of breath or difficulty breathing<br>Vomiting<br>Diarrhea ($\geq$3 looser than normal stools/24hr period)<br>Muscle aches<br>New loss in sense of smell or taste<br>Headache (new onset or worsening headache)<br>Other (fill)
24.	When did these symptoms start? (estimate as best as possible)	mm/dd
		Don’t know
25.	Did you seek healthcare for these symptoms?	Yes<br>No<br>Don’t know
26.	Were you hospitalized for COVID-19 illness?	Yes<br>No<br>Don’t know
27.	Do you live in a single-family home or multiunit housing (like an apartment)?	Single family<br>Multi-unit<br>Other (fill)
28.	Number of household members currently in the residence including yourself (resident, family, live-in staff, roommates, and long-term visitors)	Specify (scroll)
29.	What is your height?	Feet<br>Inches
30.	What is your weight?	Weight in pounds
31.1	Do you have any of the following chronic medical conditions?	Diabetes<br>Hypertension (high blood pressure)

Question no.	Questionnaire item	Response categories
		Chronic heart disease<br>Chronic kidney disease<br>Chronic liver disease<br>Asthma<br>COPD/emphysema/chronic bronchitis<br>Immunosuppressive condition (e.g., HIV, autoimmune disease)<br>Immune-weakening medication or therapy (e.g., cancer treatment)
32.	Since March 1, have you been or are you currently pregnant? (only when sex at birth is female)	Yes<br>No<br>Don’t know

*APRN, advanced practice registered nurse; BiPAP, bilevel positive airway pressure; CDC, Centers for Disease Control and Prevention; CNA, certifying nursing assistant; COPD, chronic obstructive pulmonary disease; COVID-19, coronavirus disease; CPAP, continuous positive airway pressure; DO, doctor of osteopathy; LPN, licensed practical nurse; MD, medical doctor; NP, nurse practitioner; PA, physician assistant; RN, registered nurse; SARS-CoV-2, severe acute respiratory syndrome coronavirus 2.

Appendix Table 2. Stepwise multivariable model for odds ratios of seropositivity for severe acute respiratory syndrome coronavirus 2 IgG in study of first responders and public safety personnel, New York City, New York, USA, May 18–July 2, 2020*

	Unadjusted model, n = 19,909	Adjusted model 1, n = 19,904	Adjusted model 2, n = 19,904	Adjusted model 3, n = 19,904	Adjusted model 4, n = 19,904
Characteristic	OR (95% CI)	OR (95% CI)	OR (95% CI)	OR (95% CI)	OR (95% CI)
Sex					
M	Referent	Referent	Referent	Referent	Referent
F	1.20 (1.10–1.30)	1.02 (0.93–1.11)	0.94 (0.86–1.03)	0.89 (0.81–0.98)	0.88 (0.78–0.97)
Age group, y					
18–24	Referent	Referent	Referent	Referent	Referent
25–34	0.70 (0.59–0.83)	0.66 (0.56–0.79)	0.68 (0.57–0.81)	0.78 (0.65–0.94)	0.79 (0.66–0.95)
35–44	0.49 (0.41–0.58)	0.46 (0.39–0.54)	0.53 (0.44–0.63)	0.61 (0.51–0.74)	0.63 (0.52–0.76)
45–59	0.50 (0.42–0.59)	0.47 (0.39–0.56)	0.56 (0.47–0.67)	0.61 (0.50–0.74)	0.63 (0.52–0.77)
60–64	0.50 (0.37–0.68)	0.48 (0.36–0.66)	0.58 (0.42–0.79)	0.56 (0.41–0.78)	0.59 (0.42–0.82)
>65	0.43 (0.26–0.72)	0.41 (0.24–0.68)	0.46 (0.27–0.78)	0.45 (0.26–0.77)	0.47 (0.27–0.81)
Race or ethnicity					
Non-Hispanic White	Referent	Referent	Referent	Referent	Referent
Hispanic or Latino	1.59 (1.46–1.73)	1.54 (1.42–1.68)	1.30 (1.19–1.42)	1.31 (1.20–1.44)	1.29 (1.17–1.42)
Non-Hispanic Asian	1.16 (1.01–1.33)	1.17 (1.02–1.35)	1.08 (0.93–1.25)	1.11 (0.95–1.29)	1.10 (0.94–1.28)
Non-Hispanic Black	1.91 (1.73–2.10)	1.93 (1.75–2.14)	1.67 (1.50–1.87)	1.52 (1.35–1.71)	1.50 (1.33–1.68)
Other	1.15 (0.91–1.46)	1.19 (0.94–1.50)	1.02 (0.80–1.30)	0.98 (0.77–1.25)	0.97 (0.76–1.24)
Decline to answer	0.99 (0.86–1.13)	1.06 (0.92–1.21)	0.98 (0.84–1.13)	0.95 (0.82–1.10)	0.94 (0.81–1.08)
Weight status					
Underweight or normal weight	Referent	Referent	Referent	Referent	Referent
Overweight	1.00 (0.91–1.10)	1.07 (0.97–1.18)	1.06 (0.95–1.16)	1.07 (0.97–1.19)	1.08 (0.97–1.19)
Obese	1.07 (0.97–1.18)	1.13 (1.02–1.25)	1.12 (0.99–1.23)	1.12 (1.01–1.25)	1.13 (1.01–1.26)
Severely obese	1.36 (1.10–1.68)	1.42 (1.15–1.76)	1.39 (1.10–1.71)	1.31 (1.04–1.64)	1.31 (1.05–1.65)
Housing					
Single family	Referent		Referent	Referent	Referent
Multiunit	1.33 (1.24–1.42)		1.03 (0.95–1.12)	0.99 (0.91–1.07)	0.99 (0.91–1.08)
Residence borough					
Staten Island	Referent		Referent	Referent	Referent
Bronx	1.64 (1.43–1.88)		1.18 (0.98–1.42)	1.27 (1.06–1.54)	1.27 (1.06–1.54)
Brooklyn	1.58 (1.40–1.78)		1.24 (1.06–1.45)	1.25 (1.07–1.47)	1.25 (1.06–1.46)
Manhattan	1.10 (0.93–1.30)		1.05 (0.86–1.29)	1.16 (0.94–1.44)	1.17 (0.95–1.45)
Queens	1.38 (1.23–1.55)		1.12 (0.96–1.30)	1.20 (1.03–1.40)	1.20 (1.03–1.40)
Outside New York City	0.91 (0.82–1.02)		0.79 (0.68–0.91)	0.88 (0.76–1.01)	0.89 (0.77–1.02)
Exposure to a person with COVID-19 (Referent = not exposed)					
Household member	3.77 (3.44–4.13)		3.41 (3.10–3.76)	3.51 (3.19–3.87)	3.52 (3.19–3.87)
Coworker	1.16 (1.07–1.25)		0.97 (0.90–1.05)	1.03 (0.95–1.12)	1.05 (0.97–1.15)
Patient	1.36 (1.27–1.46)		1.13 (1.05–1.22)	0.90 (0.82–0.98)	0.88 (0.80–0.97)
Other person	1.41 (1.32–1.51)		1.15 (1.07–1.25)	1.21 (1.11–1.31)	1.22 (1.13–1.32)
Workplace borough					
Staten Island	Referent		Referent	Referent	Referent
Bronx	1.65 (1.37–1.97)		1.26 (1.17–1.83)	1.27 (1.01–1.59)	1.27 (1.01–1.60)
Brooklyn	1.43 (1.21–1.71)		1.46 (1.03–1.54)	1.27 (1.03–1.55)	1.28 (1.04–1.57)
Manhattan	1.12 (0.94–1.34)		1.26 (0.90–1.38)	1.11 (0.90–1.38)	1.11 (0.90–1.38)
Queens	1.30 (1.09–1.55)		1.11 (1.01-1.56)	1.08 (0.87–1.35)	1.08 (0.87–1.35)
Occupation					
Police	Referent			Referent	Referent

Characteristic	Unadjusted model, n = 19,909	Adjusted model 1, n = 19,904	Adjusted model 2, n = 19,904	Adjusted model 3, n = 19,904	Adjusted model 4, n = 19,904
	OR (95% CI)	OR (95% CI)	OR (95% CI)	OR (95% CI)	OR (95% CI)
Correctional staff	2.75 (2.39–3.16)			2.55 (2.18–2.98)	2.55 (2.18–2.99)
Emergency medical technician	2.65 (2.35–2.97)			2.51 (2.19–2.88)	2.29 (1.95–2.69)
Traffic officer	1.98 (1.52–2.57)			2.23 (1.68–2.97)	2.06 (1.55–2.74)
Police dispatcher	2.53 (1.74–3.69)			2.04 (1.37–3.04)	1.95 (1.31–2.91)
Paramedic	1.92 (1.66–2.23)			2.07 (1.76–2.44)	1.90 (1.57–2.29)
Security guard	1.87 (1.39–2.52)			1.78 (1.29–2.46)	1.68 (1.21–2.32)
Dispatcher (Fire or EMS)	1.81 (1.40–2.34)			1.64 (1.25–2.14)	1.54 (1.17–2.02)
Firefighter	1.15 (1.05–1.25)			1.38 (1.25–1.53)	1.30 (1.15–1.46)
Other direct patient care providers†	1.12 (0.83–1.51)			1.37 (0.99–1.91)	1.32 (0.94–1.84)
Firefighter/medical first responder	0.99 (0.84–1.18)			1.12 (0.93–1.34)	1.04 (0.85–1.26)
Medicolegal death investigator	0.51 (0.33–0.79)			0.66 (0.42–1.03)	0.64 (0.41–1.00)
Aerosol-generating procedure for COVID-19 patient					
0 times	Referent				Referent
1–5 times	1.25 (1.14–1.37)				1.02 (0.91–1.14)
6–10 times	1.55 (1.34–1.79)				1.05 (0.89–1.24)
11–25 times	1.50 (1.25–1.80)				0.96 (0.78–1.17)
>25 times	1.77 (1.49–2.10)				1.02 (0.83–1.24)
Not applicable	1.11 (1.02–1.20)				1.06 (0.97–1.16)
Use of PPE within 6 feet of a person with COVID-19					
Gowns all the time	1.20 (1.11–1.29)				1.00 (0.90–1.10)
Gloves all the time	1.46 (1.36–1.56)				1.19 (1.06–1.33)
Eye protection all the time	1.36 (1.23–1.50)				1.03 (0.91–1.18)
Surgical facemask all the time	1.39 (1.30–1.49)				1.07 (0.98–1.17)
N95 respirator all the time	1.29 (1.20–1.39)				0.98 (0.88–1.09)
PAPR all the time	1.44 (1.20–1.72)				1.15 (0.94–1.42)

*Bold indicates significance. COVID-19, coronavirus disease; EMS, emergency medical service; OR, odds ratio; PAPR, powered air-purifying respirator; PPE, personal protective equipment.
†Other direct patient care providers include nurses, nurse assistants, physicians, midlevel clinicians, respiratory therapists, occupational therapists, speech therapists, physical therapists, therapy aides, and dentists.

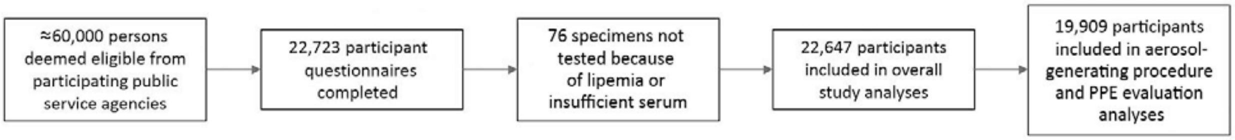

Appendix Figure 1. Flowchart of eligible participants and analytic sample in study of severe acute respiratory syndrome coronavirus 2 antibodies in public safety personnel, New York City, New York, USA, May 18–July 2, 2020. PPE, personal protective equipment.

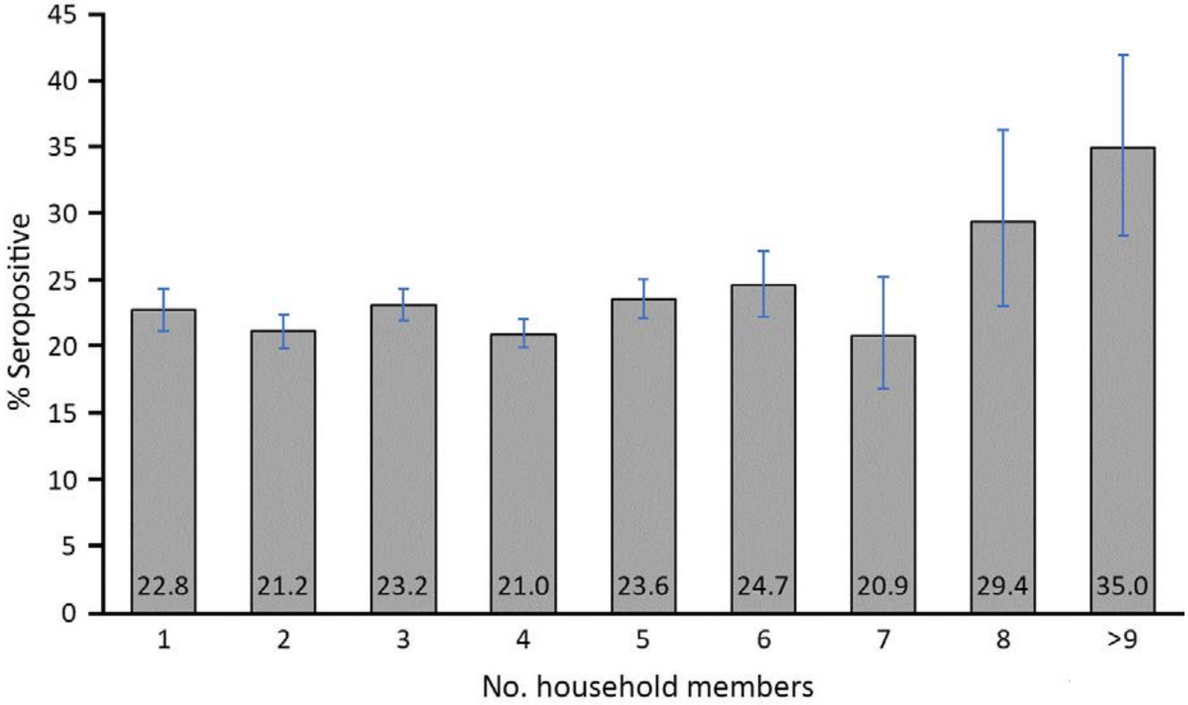

Appendix Figure 2. Percent seropositive for severe acute respiratory syndrome coronavirus 2 IgG by number of current household members in study of public safety personnel, New York City, New York, USA, May 18–July 2, 2020.

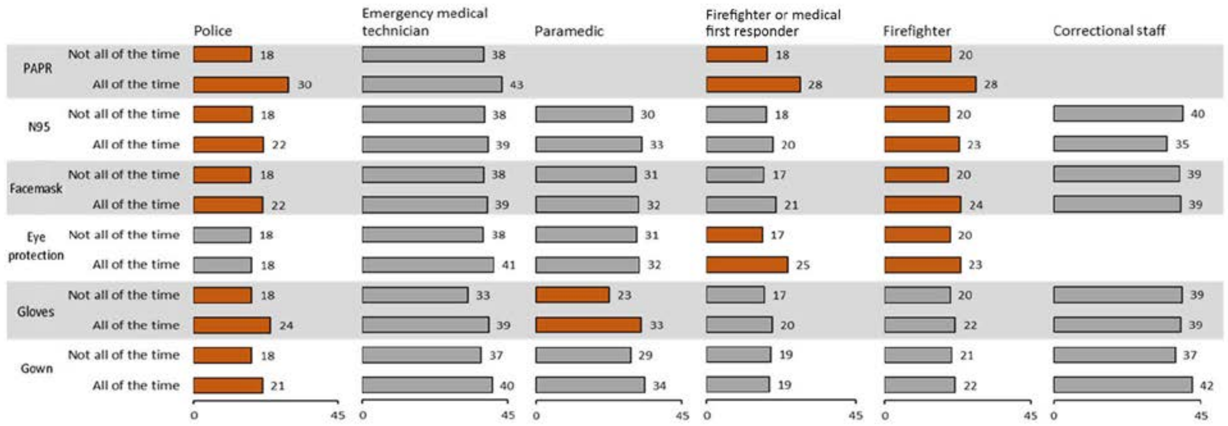

Appendix Figure 3. Percent seropositive for severe acute respiratory syndrome coronavirus 2 IgG for selected occupations, by personal protective equipment use, in study of public safety personnel, New York City, New York, USA, May 18–July 2, 2020. Red bars: Lower bound of the 95% CI for percentage positive in “all of the time” group is above the upper bound of the 95% CI for percentage positive in “not all of the time” group. Participants who reported “not applicable” were excluded. PAPR, powered air-purifying respirator.
